# Pre-treatment with the angiotensin receptor 1 blocker losartan protects renal blood flow and oxygen delivery after propofol-induced hypotension in pigs

**DOI:** 10.1038/s41598-020-74640-6

**Published:** 2020-10-21

**Authors:** Stephanie Franzén, Robert Frithiof

**Affiliations:** Department of Surgical Sciences, Anaesthesiology and Intensive Care, Uppsala University, Uppsala University Hospital, Entrance 78, 1st floor, 75185 Uppsala, Sweden

**Keywords:** Kidney, Hypertension, Kidney, Acute kidney injury, Experimental models of disease, Medical research, Preclinical research

## Abstract

Hypotensive events are strongly correlated to the occurrence of perioperative acute kidney injury, but the underlying mechanisms for this are not completely elucidated. We hypothesised that anaesthesia-induced hypotension causes renal vasoconstriction and decreased oxygen delivery via angiotensin II-mediated renal vasoconstriction. Pigs were anaesthetised, surgically prepared and randomised to vehicle/losartan treatment (0.15 mg*kg^−1^). A deliberate reduction in arterial blood pressure was caused by infusion of propofol (30 mg*kg^−1^) for 10 min. Renal function and haemodynamics were recorded 60 min before and after hypotension. Propofol induced hypotension in all animals (*p* < 0.001). Renal blood flow (RBF) and renal oxygen delivery (RDO_2_) decreased significantly regardless of treatment but more so in vehicle-treated compared to losartan-treated (*p* = 0.001, *p* = 0.02, respectively). During recovery RBF and RDO_2_ improved to a greater extent in the losartan-treated compared to vehicle-treated (+ 28 ml*min^−1^, 95%CI 8–50 ml*min^−1^, *p* = 0.01 and + 3.1 ml*min^−1^, 95%CI 0.3–5.8 ml*min^−1^, *p* = 0.03, respectively). Sixty minutes after hypotension RBF and RDO_2_ remained depressed in vehicle-treated, as renal vascular resistance was still increased (*p* < 0.001). In losartan-treated animals RBF and RDO_2_ had normalised. Pre-treatment with losartan improved recovery of renal blood flow and renal oxygen delivery after propofol-induced hypotension, suggesting pronounced angiotensin II-mediated renal vasoconstriction during blood pressure reductions caused by anaesthesia.

## Introduction

Acute kidney injury (AKI) is a common surgical complication. Approximately 8–10% of patients develop AKI after general surgery^1^ and in cardiac and vascular surgery the incidence is even higher (10–40%)^2–4^. AKI is defined as rapidly decreasing renal function graded by changes in serum creatinine levels and/or urine output^5,6^.

The kidneys receive 20–25% of cardiac output during normal conditions^7^, but are highly susceptible to ischemia due to heterogeneity in oxygenation^8^. Intraoperative hypotension and hypovolemia may result in renal ischemia and are known causes of AKI^9^. Hypotension is a frequent complication to anaesthesia since most agents used to induce anaesthesia cause vasodilation and infrequently reductions in cardiac output^10^. It has been described that even short (less than 5 min) reductions in mean arterial pressure (MAP) below 55 mmHg is independently associated with perioperative AKI^11,12^. An acute decrease in MAP outside renal autoregulation reduces renal oxygen delivery (RDO_2_). Furthermore, unloading baroreceptors increase sympathetic nerve activity and release of endogenous vasoactive hormones, such as catecholamines, renin and angiotensin-II (Ang-II), vasopressin and aldosterone^13–15^. Although this may aid in restoring MAP it theoretically causes a prolonged renal vasoconstriction, even after blood volume and/or pressure is restored.

Angiotensin converting enzyme inhibitors (ACEi) and angiotensin-II type-1 receptor blockers (ARB’s) are common treatments for hypertension^16^. It is also well accepted that high levels of Ang-II may cause renal damage and that inhibition of Ang-II is renoprotective in early chronic kidney disease^17–19^. These medications are often withheld prior to surgery to avoid perioperative hypotension^20^. However, Ang-II inhibition during anaesthesia may have beneficial renal effects.

We hypothesise that Ang-II released by propofol-induced hypotension may cause renal ischemia by reducing RDO_2_ due to prolonged renal vasoconstriction. To investigate this, we studied the effects of losartan (an intravenously administered ARB) on RBF, RDO_2_ and short-term renal function in pigs subjected to an anaesthesia-induced hypotensive event.

## Results

**Figure 1:**
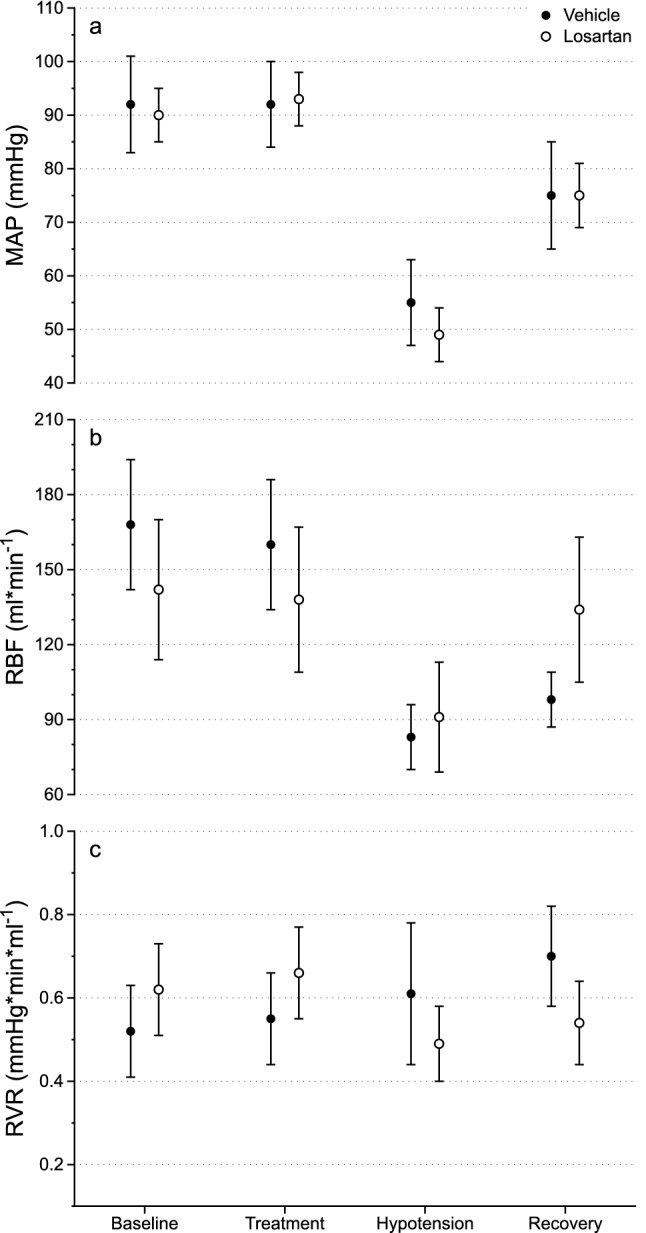
(**a**) MAP, (**b**) RBF and (**c**) RVR at baseline, treatment, hypotension and recovery in vehicle- (n = 11, black) and losartan-treated (n = 11, white) pigs. Data is displayed as mean ± 95%CI. Please refer to the main text for statistical analyses.

Mean arterial pressure (MAP) was similar in both groups at baseline (vehicle: 93 ± 9 mmHg; losartan: 90 ± 9 mmHg, *p* = 0.64, Fig. 1a) and remained unchanged by the treatment per se (vehicle: 92 ± 8 mmHg, *p* = 0.93; losartan: 93 ± 6 mmHg, *p* = 0.45, Fig. 1a). During the propofol-induced hypotensive event MAP was reduced by 38 mmHg in vehicle-treated (95%CI 30–45 mmHg, *p* < 0.001, Fig. 1a) and 42 mmHg in losartan-treated (95%CI 34–50 mmHg, *p* < 0.001, Fig. 1a) without significant intergroup difference. Recovery for 60 min improved MAP by 20 mmHg in vehicle-treated (95%CI 9–31 mmHg, *p* = 0.001, Fig. 1a) and 26 mmHg in losartan-treated (95%CI 15–37 mmHg, *p* < 0.001, Fig. 1a), with no significant intergroup difference.

At baseline renal blood flow (RBF) did not differ significantly between groups (vehicle: 168 ± 26 ml*min^−1^, losartan: 142 ± 28 ml*min^−1^, *p* = 0.18, Fig. 1b). Treatment had no effect on RBF in any of the groups (vehicle: 160 ± 26 ml*min^−1^, *p* = 0.09; losartan: 138 ± 29 ml*min^−1^, *p* = 0.81, Fig. 1b). However, RBF was significantly reduced by propofol and this effect was more pronounced in the vehicle-treated animals compared to losartan-treated (− 36 ml*min^−1^, 95%CI 2–70 ml*min^−1^, *p* = 0.001, Fig. 1b). During recovery, RBF improved to a greater extent in the losartan-treated pigs compared to vehicle-treated (+ 28 ml*min^−1^, 95%CI 8–50 ml*min^−1^, *p* = 0.01, Fig. 1b). RBF was still decreased in vehicle-treated animals 60 min after propofol was administered (98 ± 11 ml*min^−1^, *p* < 0.001, Fig. 1b). However, in the losartan group RBF had returned to baseline (134 ± 29 ml*min^−1^, *p* = 0.47, Fig. 1b).

Renal vascular resistance (RVR) did not differ between groups at baseline (vehicle: 0.52 ± 0.11, losartan: 0.62 ± 0.11 mmHg*min*ml^−1^, *p* = 0.18, Fig. 1c). It also remained unchanged by the treatment (vehicle: 0.55 ± 0.11, *p* = 0.36; losartan: 0.66 ± 0.11 mmHg*min*ml^−1^, *p* = 0.40, Fig. 1c). Propofol caused a greater increase in RVR in pigs treated with vehicle compared to losartan (0.2 mmHg*min*ml^−1^, 95%CI 0.02–0.4 mmHg*min*ml^−1^, *p* = 0.036, Fig. 1c). Vehicle-treated pigs also had significantly increased RVR after recovery compared to baseline (0.70 ± 0.12 mmHg*min*ml^−1^, *p* < 0.001, Fig. 1c) whereas losartan-treated had significantly decreased RVR compared to baseline (0.54 ± 0.10 mmHg*min*ml^−1^, *p* = 0.03, Fig. 1c). However, no significant differences between the groups was demonstrated after 60-min recovery (vehicle: 0.61 ± 0.17, losartan: 0.49 ± 0.09 mmHg*min*ml^−1^, *p* = 0.07, Fig. 1c).

### Renal oxygenation

**Figure 2:**
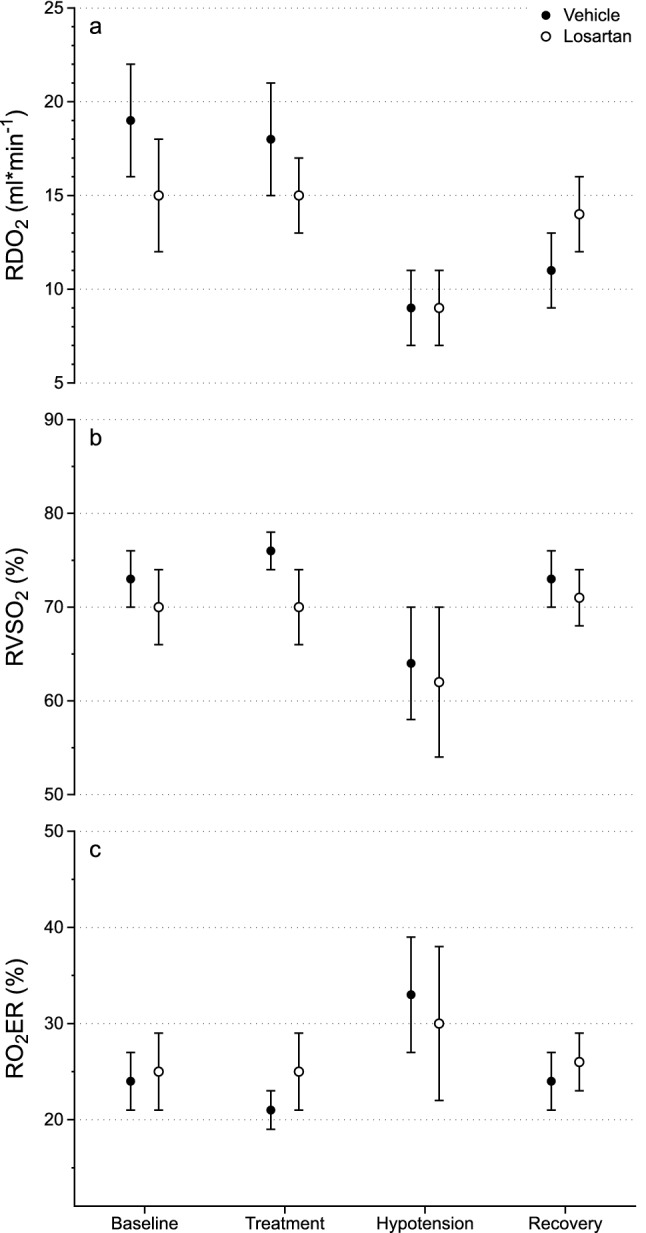
(**a**) RDO_2_, (**b**) RVSO_2_ and (**c**) RO_2_ER over time at baseline, treatment, hypotension and recovery in vehicle- (n = 11, black) and losartan-treated (n = 11, white) pigs. Data is displayed as mean ± 95%CI. Please refer to the main text for statistical analyses.

Renal oxygen delivery (RDO_2_) was, as expected, affected in a similar manner as RBF. Baseline RDO_2_ was not significantly different between the groups (vehicle: 19 ± 3 ml*min^*−*1^, losartan: 15 ± 3 ml*min^*−*1^, *p* = 0.06, Fig. 2a) and treatment did not affect RDO_2_ in either group (vehicle: 18 ± 3 ml*min^*−*1^, *p* = 0.21; losartan: 15 ± 2 ml*min^*−*1^, *p* = 0.72, Fig. 2a). The propofol-induced hypotensive event caused a more pronounced decrease in RDO_2_ in vehicle-treated pigs (4.5 ml*min^*−*1^, 95%CI 0.6–8.3 ml*min^*−*1^, *p* = 0.02, Fig. 2a) compared to losartan-treated. RDO_2_ recovery after hypotension was also improved by losartan compared to vehicle (+ 3.1 ml*min^*−*1^, 95%CI 0.3–5.8 ml*min^*−*1^, *p* = 0.03, Fig. 2a). Compared to baseline vehicle-treated pigs had significantly lower RDO_2_ after recovery (11 ± 2 ml*min^*−*1^, *p* < 0.001, Fig. 2a). Losartan-treated pigs made a full recovery with regards to RDO_2_ (14 ± 2 ml*min^*−*1^, *p* = 0.35, Fig. 2a).

Renal Vein Oxygen Saturation (RVSO_2_) was not different between groups at baseline (vehicle: 73 ± 3%, losartan: 70 ± 4%, *p* = 0.29, Fig. 2b). Losartan had no effect on this parameter (70 ± 4%, *p* = , Fig. 2b). However, in vehicle-treated pigs RVSO_2_ increased by 3% (96%CI 0–5%, *p* = 0.02). Propofol-induced hypotension decreased RVSO_2_ in both vehicle- and losartan-treated (9% 95%CI 2–16%, *p* = 0.02 and 8% 95%CI 1–15%, *p* = 0.03, respectively, Fig. 2b), with no intergroup differences. Both groups increased RVSO_2_ after recovery compared to hypotension (vehicle: 9% 95%CI 2–15%, *p* < 0.009; losartan: 9% 96%CI 2–15, *p* = 0.01, Fig. 2b), with no significant intergroup difference.

Renal oxygen extraction rate (RO_2_ER) was not different at baseline (vehicle: 24 ± 3%, losartan: 25 ± 4%, *p* = 0.20, Fig. 2c). Vehicle-treated decreased RO_2_ER during the treatment period (3% 95%CI 0–5%, *p* = 0.02, Fig. 2c) meanwhile losartan-treated remined unchanged (25 ± 4%, *p* = 0.49, Fig. 2c). Hypotension increased RO_2_ER in both groups by 8% in vehicle-treated (95%CI 1–16%, *p* = 0.02, Fig. 2c) and 8% in losartan-treated (95%CI 0–15%, *p* = 0.04, Fig. 2c) without intergroup difference. After recovery, RO_2_ER in both groups normalised (vehicle: 24 ± 3%, *p* = 0.81; losartan: 26 ± 3%, *p* = 0.51, Fig. 2c).

Renal oxygen consumption (RVO_2_) did not differ between groups at baseline (vehicle: 5.0 ± 0.9 mmol*min^−1^, losartan: 3.3 ± 1.3 mmol*min^−1^, *p* = 0.32, Table 1). In vehicle-treated pigs RVO_2_ decreased significantly (− 0.9 mmol*min^−1^) during the treatment period (95%CI 0.1–1.8 mmol*min^−1^, *p* = 0.04, Table 1) whereas in losartan-treated pigs RVO_2_ did not change (3.4 ± 1.5 mmol*min^−1^, *p* = 0.98, Table 3). Hypotension decreased RVO_2_ even further in the vehicle-treated pigs (− 0.9 mmol*min^−1^ 95%CI 0.1–1.8 mmol*min^−1^, *p* = 0.02, Table 1). In losartan-treated pigs RVO_2_ increased compared to baseline as response to propfol (+ 0.9 mmol*min^−1^ 95%CI 0.1–1.8 mmol*min^−1^, *p* = 0.04, Table 1). During recovery RVO_2_ remained depressed in pigs treated with vehicle (2.6 ± 0.6 mmol*min^−1^, *p* = 0.2) but not losartan (3.2 ± 0.7 mmol*min^−1^, *p* = 0.26, Table 1).

**Table 1:** Renal Lactate (R-Lac), Renal Oxygen Consumption (RVO_2_), plasma creatinine (P-Cr), creatinine clearance (Cr-Clearance), urine output and urinary sodium excretion (USE) in vehicle- and losartan-treated pigs during the treatment period and recovery period in vehicle- and losartan-treated.

		Vehicle	Losartan
R-Lac (mmol*l^−1^)	*Baseline*	1.3 ± 0.1	1.6 ± 0.2^†^
	*Treatment*	1.3 ± 0.2	1.5 ± 0.2
	*Hypotension*	1.5 ± 0.2	1.8 ± 0.3
	*Recovery*	1.4 ± 0.3*	1.5 ± 0.3*
RVO_2_ (mmol*min^−1^)	*Baseline*	5.0 ± 0.9	3.3 ± 1.3
	*Treatment*	4.0 ± 0.8*	3.4 ± 1.5
	*Hypotension*	3.0 ± 0.4*	2.7 ± 0.8*
	*Recovery*	2.6 ± 0.6	3.2 ± 0.7
P-Cr (µmol*l^−1^)	*Treatment*	66 ± 11	64 ± 9
	*Recovery*	65 ± 11	67 ± 10
Cr-Clearance (mmol*min^−1^)	*Treatment*	77 ± 11	60 ± 9
	*Recovery*	68 ± 15	55 ± 16
Urine Output (ml*min^−1^)	*Treatment*	1.5 ± 0.5	1.2 ± 0.9
	*Recovery*	1.2 ± 0.2	1.4 ± 1.1
USE (mmol*min^−1^)	*Treatment*	110 ± 40	123 ± 59
	*Recovery*	77 ± 29	136 ± 73

*Denotes within-subject effects: timepoint vs previous timepoint, ^†^denotes between-subject effects at that timepoint.

Renal Lactate levels (R-Lac) was significantly higher in losartan-treated at baseline (vehicle: 1.3 ± 0.1 mmol*l^−1^, losartan: 1.6 ± 0.2 mmol*l^−1^, *p* = 0.01, Table 1). Vehicle- and losartan-treated pigs remained unchanged by treatment with regards to R-Lac (1.3 ± 0.2 mmol*l^−1^, *p* = 0.85 and 1.5 ± 0.2 mmol*l^−1^, *p* = 0.31, respectively, Table 1) and by hypotension (1.5 ± 0.2 mmol*l^−1^, *p* = 0.11 and 1.8 ± 0.3 mmol*l^−1^, *p* = 0.27, respectively, Table 1). After the 60-min recovery-period R-Lac was unchanged in vehicle-treated pigs (1.3 ± 0.2 mmol*l^−1^, *p* = 0.35, Table 1) but had decreased in losartan-treated pigs compared to hypotension (0.25 mmol*l^−1^ 95%CI 0.1–0.4 mmol*l^−1^, *p* = 0.007, Table 1).

### Renal parameters

Plasma creatinine, creatinine clearance, urine output and urinary sodium excretion did not change significantly as an effect of losartan treatment or propofol (Table 1).

### Systemic perfusion

Carotid Blood Flow (CBF) was not significantly different at baseline between the groups (vehicle: 148 ± 19 ml*min^−1^, losartan: 122 ± 23 ml*min^−1^, *p* = 0.10, Table 2), and treatment had no effect on CBF in either group (vehicle: 152 ± 20 ml*min^−1^, *p* = 0.61; losartan: 129 ± 22 ml*min^−1^, *p* = 0.43, Table 2). Hypotension did not significantly change CBF in either vehicle-treated (159 ± 30 ml*min^−1^, *p* = 0.50, Table 2) nor losartan-treated (113 ± 18 ml*min^−1^, *p* = 0.57, Table 3). CBF remained unchanged in vehicle-treated pigs during recovery (175 ± 32 ml*min^−1^, *p* = 0.44, Table 2) but increased in losartan-treated animals compared to hypotension (+ 65 ml*min^−1^ 95%CI 22–107 ml*min^−1^, *p* = 0.004, Table 2).

**Table 2:** Carotid blood flow (CBF), cardiac output (CO) and heart rate (HR) in vehicle-treated and losartan-treated pigs during baseline, treatment, hypotension and recovery.

		Vehicle	Losartan
CBF (ml*min^−1^)	*Baseline*	151 ± 28	123 ± 42
	*Treatment*	161 ± 35	125 ± 33
	*Hypotension*	161 ± 56	103 ± 30
	*Recovery*	174 ± 64	175 ± 62*
CO (ml*min^−1^)	*Baseline*	2.9 ± 1.0	3.1 ± 0.7
	*Treatment*	3.3 ± 0.8	3.3 ± 0.6
	*Hypotension*	3.1 ± 1.2	2.7 ± 1.1
	*Recovery*	3.6 ± 1.2	3.8 ± 0.6*
HR (beats*min^−1^)	*Baseline*	99 ± 19	103 ± 22
	*Treatment*	105 ± 15	102 ± 19
	*Hypotension*	103 ± 15	102 ± 22
	*Recovery*	106 ± 19	114 ± 17*

*Denotes within-subject effects: timepoint vs previous timepoint.

**Table 3:** Central venous pressure (CVP), pulmonary artery pressure (PAP), pulmonary wedge pressure (PWP) and renal venous pressure (RVP) in vehicle-treated and losartan-treated pigs during baseline, treatment, hypotension and recovery.

		Vehicle	Losartan
CVP (mmHg)	*Baseline*	6 ± 1	6 ± 1
	*Treatment*	6 ± 1	6 ± 1
	*Hypotension*	6 ± 1	6 ± 1
	*Recovery*	5 ± 1*	5 ± 1*
PAP (mmHg)	*Baseline*	20 ± 2	17 ± 1
	*Treatment*	19 ± 1	18 ± 1
	*Hypotension*	17 ± 1*	16 ± 1*
	*Recovery*	19 ± 1*	21 ± 1*
PWP (mmHg)	*Baseline*	8 ± 1	7 ± 1
	*Treatment*	8 ± 1	8 ± 1
	*Hypotension*	9 ± 1	8 ± 1
	*Recovery*	8 ± 1	8 ± 1
RVP (mmHg)	*Baseline*	11 ± 1	9 ± 1
	*Treatment*	10 ± 1	8 ± 1
	*Hypotension*	10 ± 1	8 ± 1
	*Recovery*	9 ± 1	9 ± 1

*Denotes within-subject effects: timepoint vs previous timepoint.

Cardiac Output (CO) was not different at baseline when comparing the two groups (vehicle: 3.1 ± 0.6 ml*min^−1^, losartan: 3.2 ± 0.4 ml*min^−1^, *p* = 0.75, Table 2). Treatment had no significant effect on CO in either group (vehicle: 3.3 ± 0.4 ml*min^−1^, *p* = 0.33; losartan: 3.4 ± 0.4 ml*min^−1^, *p* = 0.35, Table 2). Propofol-induced hypotension did not affect CO in either group (vehicle: 3.3 ± 0.8 ml*min^−1^, *p* = 0.52; losartan: 3.1 ± 0.7 ml*min^−1^, *p* = 0.39, Table 3). After recovery CO was not significantly different in vehicle-treated compared to hypotension (4.0 ± 0.7 ml*min^−1^, *p* = 0.07, Table 2). However losartan increased CO by 1.0 ml*min^−1^ during recovery compared to hypotension (95%CI 0.3–1.8 ml*min^−1^, *p* = 0.01, Table 2).

Heart Rate (HR) did not differ between groups at baseline (vehicle: 95 ± 11 bpm, losartan: 102 ± 16 bpm, *p* = 0.47, Table 3) and was not changed by the treatment (vehicle: 102 ± 9 bpm, *p* = 0.14; losartan: 99 ± 11 bpm, *p* = 0.42, Table 2) or hypotension (vehicle: 104 ± 8 bpm, *p* = 0.21; losartan: 100 ± 12 bpm, *p* = 0.68, Table 2). During recovery HR was not significantly changed in vehicle-treated (112 ± 12 bpm, *p* = 0.10, Table 3), However losartan increased HR by 13 bpm (95%CI 1–26 bpm, *p* = 0.04, Table 2).

CVP decreased by 2 mmHg (95%CI 0–3 mmHg, *p* = 0.04, Table 3) after recovery compared to hypotension without intergroup difference.

PAP decreased by 2 mmHg (95%CI 0–4 mmHg, *p* = 0.007, Table 3) during hypotension compared to baseline with no intergroup difference. PAP then increased after recovery compared to hypotension without intergroup difference (3 mmHg 95%CI 2–5 mmHg, *p* < 0.001, Table 3).

PWP and RVP was not significantly changed during the experimental protocol.

## Discussion

This study was conducted to evaluate the acute effects of systemic Ang-II antagonism on renal perfusion, oxygenation and function after an anaesthesia-induced hypotensive event. The main finding was that losartan improved recovery of RBF and RDO_2_ after a hypotensive dose of propofol. The data obtained indicates that RBF may be impaired for a considerable amount of time after hypotension even though usually monitored clinical parameters (i.e. MAP, HR, CO, plasma-lactate or urine output) remained unchanged or have normalised.

Perioperative AKI has been described as an under-recognised problem with few available preventive treatment strategies^21^. In a majority of cases the underlying mechanism causing renal dysfunction is unknown^22,23^. Propofol is an agent frequently used to induce general anaesthesia. It is also a potent vasodilator which entails that the most common side-effect is hypotension^10^.

Ang-II causes powerful systemic vasoconstriction through Ang-II receptor 1 (AT_1_) binding. This effect is regionally differentiated in that the renal circulation constricts more in comparison to other vascular beds^24^. Plasma levels of Ang-II are increased by hypotension and decreases RBF and glomerular filtration rate^25^. In this study we designed an experiment to investigate if renal perfusion was impaired due to Ang-II. The aim was to mimic a clinical anaesthesia induction resulting in an unwanted but transient hypotensive event. Even if hypotension is swiftly corrected the resulting potential increase in Ang-II may cause a persistent reduction in RBF that is difficult to monitor and treat in anaesthetised patients undergoing surgery. Patients that are extra susceptible to renal ischemia, perhaps due to diabetes, hypertension, cardiovascular disease and/or prior kidney disorders may then develop AKI^1,26^. The reduction in RBF may be insufficient to cause postoperative renal dysfunction in otherwise healthy patients. Previous studies have shown detrimental renal effects of high circulating levels of Ang-II in a variety of settings^27^. A clinical study showed that inhibition of Ang-II in diabetic CKD patients had beneficial effects on renal oxygenation^28^. Furthermore, the current study indicates that Ang-II may also be a culprit in acute settings of anaesthesia-induced hypotension.

Continuous medication targeting Ang-II is common in large population of patients, such as CKD, diabetes and cardiovascular disease. These groups of patients also have higher risk for perioperative AKI. Prolonged renal vasoconstriction and decreased RDO_2_ may provoke a mismatch in O_2_ consumption and delivery ultimately leading to hypoxia^29,30^. In current clinical practice it is common procedure to withhold any ACEi/ARB’s prior to surgery. The rationale is to avoid hypotension and cardiac events^31^. However, the preoperative management of ACEi/ARB’s are extensively debated and currently the data for continuing or withholding preoperative treatment is not clear^20,32^. It was suggested over 20 years ago that hypertensive patients should continue Ang-II antagonistic treatment before surgery^33^. On the other hand, Brabant et al. demonstrated significantly higher risk for reduced arterial blood pressure and more treatment-resistant hypotensive events in patients continuing ARB’s compared to other anti-hypertensives^34^. Interestingly, a recent multicentre study demonstrated no significant differences in AKI outcome in patients with ongoing ARB’s/ACEi compared to patients withholding treatment. The potent vasoconstriction achieved by pharmacological treatment with Ang-II have also been demonstrated to have beneficial effects on blood pressure and reduced need of vasopressors during refractory shock^35^.

The reductions in RBF and increase in RVR in vehicle-treated pigs were significant 60 min after propofol was discontinued, even though MAP had more or less recovered. Losartan attenuated the renal vasoconstriction and normalised renal blood flow. Although uncertain in this study as we did not measure AngII-levels in plasma, this was likely due to inhibition of excessive release of Ang-II caused by hypotension^36^. As a result, RDO_2_ was reduced in the vehicle-treated pigs but greatly improved by losartan. It has also been shown that anaesthesia per se cause release of Ang-II^37^. Since losartan did not reduce MAP prior to hypotension the contribution of Ang-II to baseline blood pressure during anaesthesia in the current setting is likely small. The lack of pressure effect inhibiting systemic Ang-II is much like what is seen in normotensive conscious humans^38^.

RVO_2_ was decreased in the losartan-treated animals during the hypotensive event. Ang-II acts via the AT_1_-receptor to stimulate Na/K-ATPase and, in theory, thereby increasing renal oxygen consumption. This effect was not noted during the relatively short protocol executed in this study. Unfortunately oxygenation in renal tissue was not measured and it is possible that local hypoxia was present although not to the degree that it was reflected in renal vein blood gases. Furthermore, the subjects were healthy, young pigs with kidneys most likely more resistant to decreased renal blood flow than the average human patient undergoing general anaesthesia.

Other parameters for assessing renal function such as P-Cr, Cr-C, USE and urine output did not change during the course of the protocol. This is most likely due to the acute setting and that more profound renal impairment may be necessary for these markers to be altered in previously healthy individuals^39^.

Cardiovascular parameters were continuously monitored to ensure that systemic haemodynamics were not negatively affected by losartan and to identify possible mechanisms for changes in RBF. Losartan per se had no adverse effects on haemodynamics during the study protocol. Importantly, CBF remained unaffected by losartan during hypotension suggesting that losartan did not impair cerebral perfusion. Another crucial aspect is that losartan did not augment the hypotension caused by propofol. This is in agreement with the finding that MAP was affected similarly during hypovolaemia in anaesthetised pigs treated with either ACEi/ARB’s or vehicle^40^.

### Limitations

All animals in this study arrived one at the time after a short transport and was assessed by experienced lab-technicians to be calm and non-stressed at time of anaesthesia induction. A mix of male and female pigs was used to eliminate gender variability. It would have been preferable to have blinded this study to rigorise the results, however due to practical reasons this was not feasible. The acute setting limits the information provided on possible long-lasting effects of Ang-II inhibition during anaesthesia. Furthermore, the relatively short duration of ischemia in young and healthy individuals also reduce the likelihood of renal structural damage; therefore no histological samples were collected. The purpose of this study was to demonstrate the physiological effects of Ang-II blockade. However, biomarkers such as urinary kidney injury marker-1 (KIM-1) or neutrophil gelatinase-associated lipocalin (NGAL) could have been analysed in order to detect mild renal injury. Plasma levels of Ang-II were not measured in this study and because of that we cannot definitely conclude that Ang-II levels were elevated in response to hypotension. However, it has been shown in similar animal models^36^.

Finally, the present study uses a relatively small number of healthy pigs as a large animal model of the human situation. This is to enable research impossible to perform in patients, describing basic mechanisms of action. The results should be interpreted carefully and cannot be directly transferred to the clinic. Still, the prevention of renal vasoconstriction by losartan presented here merits future investigations of the renal effects of Ang-II inhibition in anesthetized human patients.

## Conclusion

The present study demonstrate that pre-treatment with losartan prevent renal vasoconstriction and improve recovery of renal blood flow and oxygen delivery after anaesthesia-induced hypotension.

## Methods

### Ethical approval and animals

All methods were carried out in accordance to relevant guidelines and regulations. Ethical approval for this study (Dnr. 5.8.18-02325/2019) was provided by the Uppsala Animal Ethics Board of the Swedish Board of Agriculture (Jordbruksverket), Sweden on March 29, 2019. Male and female Norwegian Landrace breed/Hampshire/Yorkshire pigs (24 ± 2 kg, 3–4 months old) were purchased from a local farmer in Uppsala, Sweden, to eliminate gender variability. The pigs were born on the farm and housed in group in large cages with water and food *ad* libitum. A total of 22 pigs were used; 11 vehicle-treated and 11 losartan-treated. Pigs arrived at the laboratory at 8.00 am (a 20-min truck ride in cage) one/two at the time and were randomised to treatment/control group by an ‘every other’ method.

### Anaesthesia

At the time of arrival, the pigs were weighed and sedated with an intramuscular injection of tiletamine-zolazepam (Zoletil 6 mg*kg^−1^) and xylazine (Rompun 2.2 mg*kg^−1^). After 3 min, pigs were tracheostomised and put under mechanical ventilation and given ketamine (Ketaminol 20 mg) and morphine (20 mg) in a peripheral vein in the ear. Pentobarbital (8 mg*kg^-1^*h^−1^) and morphine (0.26 mg*kg^−1^*h^−1^) dissolved in a glucose/sodium-solution (sodium chloride 2.5 mg*ml^−1^ and glucose 25 mg*ml^−1^) was given for maintenance of anaesthesia and rocuronium (Esmeron 2,5 mg*kg^−1^*h^−1^) for muscle relaxation. After finished experimental protocol, pigs were euthanised with potassium chloride.

### Surgical preparation

Peripheral vein catheters were placed in each ear for heated (38 °C) infusion of anaesthesia and Ringer’s acetate (15 mg*kg^−1^*h^−1^ for the first hour followed by 10 mg*kg^−1^*h^−1^). Mechanical ventilation was set to achieve arterial PCO_2_ 4.5–5.5 kPa with FiO_2_ 0.30. Tidal volume (TV) was started at 10 ml*kg^-1^, respiratory rate (RR) 25 and positive end-expiratory pressure (PEEP) at 5 cm H_2_O, if needed TV/RR was adjusted to maintain arterial PCO_2_ 4.5–5.5 kPa. The carotid artery on the right side was catheterised with a single lumen catheter for continuous monitoring of MAP and blood sampling. The jugular vein on the right side was catheterised with a 3 lm-catheter for cardiac output, fluid infusion and continuous monitoring of central venous pressure (CVP). Furthermore, a balloon-tipped pulmonary artery catheter (7.5F Swan-Ganz, Edwards Lifesciences, Irvine, CA) was placed into the right jugular vein and advanced into the pulmonary artery for monitoring of pulmonary arterial pressure (PAP), pulmonary wedge pressure (PWP) and blood sampling. The location of the PA-catheter was confirmed by assessing the pressure-curve on the monitor derived from the tip of the catheter. After the right atrium was reached, the catheter was carefully advanced into the pulmonary artery. To confirm correct placement, the balloon was inflated to obtain a pulmonary wedge pressure curve on the monitor. A flow probe (FSB-series 3, Transonic, Ithaca, NY) was placed around the carotid artery on the left side for monitoring of carotid blood flow (CBF). The left jugular vein was catheterised with a single lumen catheter for blood gas analysis. A suprapubic catheter (Foley no. 8. A Datex-Ohmeda S/5 monitor, Madison, WI) was placed in the bladder for urine collection. The pigs were then turned to lay on their right side. A 10 cm incision was made from rib to hip to locate and dissect the left kidney. A flow probe (FSB-series 4) was placed around the renal artery for continuous monitoring of RBF. A single lumen catheter was then placed into the renal vein for monitoring of renal vein pressure (RVP) and blood sampling. All incisions were closed with sutures (Prolene 3.0). After surgical preparation, the pigs were allowed to recover for 45 min before the experiment commenced.

### Experimental protocol

**Figure 3:**
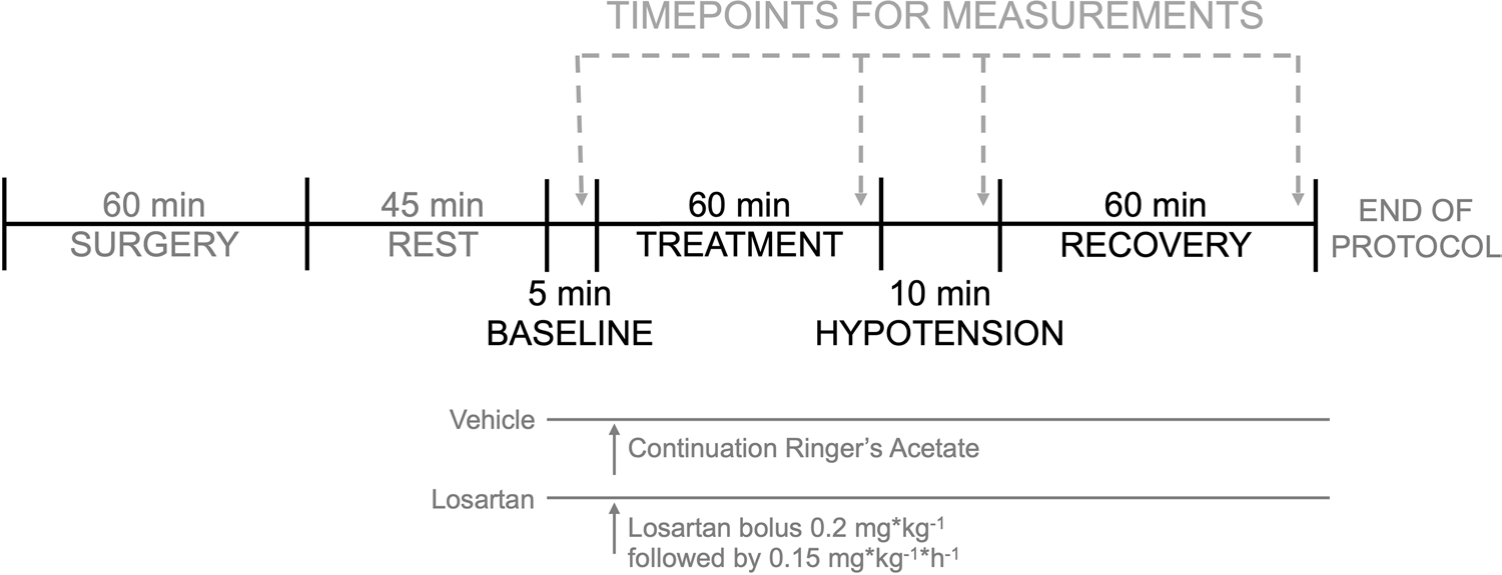
Flow chart of the experimental protocol. Pigs were sedated, surgically prepared and then allowed to recover. Baseline measurements were performed before infusion of either losartan or vehicle. The infusion continued for 60 min before hypotension was induced by administering propofol (30 mg*kg^−1^) continuously over 10 min. After the infusion of propofol was discontinued the animals were followed for an additional 60 min. The protocol was then ended, and pigs were euthanised with intravenous bolus of potassium chloride.

Pigs were block-randomised to no treatment (vehicle, n = 11) or losartan treatment (n = 11). Baseline recordings for 5 min were performed before treatment was started. An intravenous infusion of losartan was commenced with a bolus (0.2 mg*kg^−1^) followed by a continuous infusion (0.15 mg*kg^−1^*h^−1^) similar to previously reported experiments^41^. Vehicle-treated pigs received no additional infusion. The experiment was divided into four consecutive periods; baseline (5 min before drug administration), treatment (60 min with drug infusion), hypotension (10 min with propofol infusion) and recovery (60 min). Hypotension was induced by continuous infusion of propofol (30 mg*kg^−1^). At every timepoint (Fig. 3), blood was sampled for blood-gas status and analysis of creatinine, sodium and potassium. Vital parameters were noted by reading of the mechanical ventilator, the monitor for pressures and the Transonic flow-meter. In addition, arterial blood was also collected halfway through the treatment and recovery periods. Urine was continuously collected only over the 60-min periods (treatment and recovery).

Blood and urine samples were analysed by the in-house hospital laboratory at the Uppsala University Hospital (Clinical Chemistry Laboratory) to evaluate plasma and urinary creatinine (enzymatic method) and sodium/potassium levels (flame photometry). Blood gases were sampled from the carotid artery, renal vein, jugular vein and pulmonary artery to evaluate pO_2_, PCO_2_, SO_2_, haemoglobin, lactate and electrolyte levels (Radiometer Medical ApS, ABL800 FLEX, Brönshöj, Denmark). Parameters that were registered and calculated for results are MAP, RBF, renal vascular resistance (RVR), RDO_2_, CBF, cardiac output (CO), heart rate (HR), renal venous oxygen saturation (RVSO_2_), renal venous lactate (RLac), renal oxygen consumption (RVO_2_), renal oxygen extraction rate (RO_2_ER), creatinine clearance (Cr-C), plasma creatinine (P-Cr), urine output and urinary sodium excretion (USE). The cardiovascular pressures CVP, PAP, PWP was only monitored to confirm heart and pulmonary function.

### Calculations

RVR = (MAP − RVP) * RBF^−1^.

O_2_ content = (SO_2_ * haemoglobin * 1.39) + (pO_2_ * 0.003).

RDO_2_ = (RBF * arterial O_2_ content) * 1000^−1^.

RVO_2_ = ((arterial O2 content − renal venous O2 content) * RBF) * 1000^−1^.

RO_2_ER = (arterial SO_2_ − renal venous SO_2_) * arterial SO_2_^−1^.

Urine output = volume * minutes^−1^.

Cr-C = (urinary Cr * urine output) * P-Cr^−1^.

USE = urinary Na^+^ * urine output^−1^.

### Statistical analysis

All data are displayed as mean ± 95% confidence interval. The software Statistica (StatSoft, Uppsala, Sweden) was used for statistical analysis. The repeated measurements analysis of variance with 2 levels of between-subject factors and 4 levels of within-subject factors (RM 2 × 4 ANOVA) was used. Corrections for multiple comparisons were made with specific contrasts as posthoc analysis.

## Supplementary information

Supplementary file1

## Data Availability

All data generated or analysed during this study are included in this published article (and its Supplementary Information files).
