# Supplementary material for: Pre-treatment with the angiotensin receptor 1 blocker losartan protects renal blood flow and oxygen delivery after propofol-induced hypotension in pigs
Source: Sci Rep. 2020 Oct 21;10:17924. doi: 10.1038/s41598-020-74640-6 (PMC7578078; doi:10.1038/s41598-020-74640-6)
Supplement: Supplementary file 1 — Supplementary file1 [file 41598_2020_74640_MOESM1_ESM.pdf]

2020-06-18

Supplemental file

Pre-treatment with the angiotensin receptor 1 blocker losartan protects renal blood flow and oxygen delivery after propofol-induced hypotension in pigs.

Stephanie Franzén and Robert Frithiof

Additional experiments

Six pigs were added to the study.
The pigs (all 25 kg) arrived at the laboratory two at the time. At the time of arrival, the pigs were weighed and sedated with an intramuscular injection of tiletamine-zolazepam (Zoletil 6 $mg*kg^{-1}$) and xylazine (Rompun 2.2 $mg*kg^{-1}$). After 3 minutes, pigs were tracheostomized and put under mechanical ventilation and given ketamine (Ketaminol 20 mg) and morphine (20 mg) in a peripheral vein in the ear. Pentobarbital (8 $mg*kg^{-1}*h^{-1}$) and morphine (0.26 $mg*kg^{-1}*h^{-1}$) dissolved in a glucose/sodium-solution (sodium chloride 2.5 $mg*ml^{-1}$ and glucose 25 $mg*ml^{-1}$) was given for maintenance of anaesthesia and rocuronium (Esmeron 2,5 $mg*kg^{-1}*h^{-1}$) for muscle relaxation. After finished experimental protocol, pigs were euthanized with potassium chloride.

The pigs were then surgically prepared as described in the main manuscript and followed the same experimental protocol and timeline as previous experiments. Three pigs were randomized to losartan-treatment and the rest to vehicle-treatment. The new data was added to the groups and a new statistical analysis was performed in Statistica (StatSoft).
The result section and the graphs were modified according to the new results. The main conclusions remained the same.
